# The General Examination of the Child's Skin

**Published:** 1908-01-04

**Authors:** 


### Dermatology.

### THE GENERAL EXAMINATION OF THE CHILD'S SKIN.

It is sometimes said that the child's skin reacts in a manner different from that of an adult, that its various lesions resemble one another with provoking frequency, and that its eruptions are often so transitory that they may be missed altogether. There are many rashes, also, that seem to defy classification or even recognition. In the main, these statements are true, but the extent to which they influence practice varies according to one's familiarity with certain peculiarities shown by the cutaneous system of an infant. There is no golden key to the diagnosis of an eruption in a young child, and the problems presented for solution are often rendered more complex owing to the fact that we have to rely largely upon objective symptoms. In the case of infants, even among the upper classes, it is seldom possible to obtain anything like an accurate history of the onset of an eruption or what it looks like when it first appears, two points which perhaps more than anything else are of real value in arriving at a diagnosis.

### Inspection of Whole Surface Necessary.

It is never safe to assume that a widely spread eruption is similar in character to that seen upon one localised area. Thus, an eczema which is weeping upon the face may be papular over the body, fissured upon the hands, and so on. The whole cutaneous surface should be thoroughly and systematically inspected, and it is much easier to do this in a child than in an adult. Only one part of the body need be exposed at a time, and it is essential not to frighten the little patient during this process. Epilation-forceps, or even the hand-lens, should be judiciously hidden in the hand until wanted, or the child may play with them while the mother or nurse is engaged in conversation. The room and the examining hand should be pleasantly warm, and the examination should be conducted as quickly as is compatible with efficiency. A doubtful case of scalp-ringworm, involving, perhaps, a long search for one suspicious hair, will, of course, take some time; but a surface-scraping of a supposed tinea patch upon the smooth skin need not occupy more than a minute. A note as to the general condition of the whole integument should be made, especially the presence of any congenital anomaly, such as hypertrichosis, moles, or warts. There may be a tendency to xerodermia, as seen in the somewhat withered-looking skin over the hands, feet, and face, together with the intensification of the normal lines of cleavage of the skin, and a dry, lustreless condition of the epidermis as a whole. Such a skin is especially prone to be affected with eczema, and it will probably be found that there is a family history of xerodermia. The shins should be specially examined also, for here ichthyosis, an exaggerated degree of xerodermia, may be seen. Another variety of roughened skin is due to a chronic hypertrophy of the hair-follicles, which is apt to show itself in the form of circular patches upon the extensor surfaces of the limbs and trunk. Sometimes these little areas are as prickly as a nutmeg grater, in which case a small thickened spine can be observed standing out from the centre of such an enlarged follicle. The two conditions keratosis pilaris and lichen spinulosus, may be co-existent, but more commonly they are independent.

The presence of scratch-marks affords a valuable clue as to the true nature of an infantile eruption. A very young infant cannot scratch, but it can inflict quite as much damage by continually chafing and rubbing an affected part. This action may be going on in our presence, even during the examination, when of course, it is unnecessary to ask the mother if the rash itches. The papule, or rather a papular wheal, surmounted by a minute blood-crust, especially when scattered fairly thickly upon the loins, buttocks, and thighs of an infant between the ages of nine months and two years, is pretty characteristic of the very common affection known as urticaria papulosa (lichen urticatus). In older children a similar condition may be associated with chronic enlargement of the neighbouring lymphatic glands, the urticarial element being practically absent. This condition is known as prurigo, and it exists in two distinct forms, a mild and a severe. A universal eruption in a child is frequently a scabies or an eczema, both of which diseases may be complicated by a super-added impetigo.

The distribution of the lesions will help us a great deal in deciding from which of the latter complaints a child is suffering. The flexures of the joints, the folds of the neck and groin should be examined carefully for moist patches, while the fronts of the wrists, the interdigital clefts, the umbilical region, and the toes should be searched with the lens for the burrows of the itch-mite. Much crusting will, of course, favour the diagnosis of impetigo, especially if it can be ascertained that the lesions have been produced by local auto-inoculation. In a suspected syphilitic eruption of congenital origin, the palms and soles should receive special attention, for it is pretty safe to assume that if these regions be free the case is not specific, in conjunction, of course, with the absence of other signs. Much irritation is, moreover, seldom associated with a syphilide, unless the surface has become sore and abraded.

It may happen that at the time of the examination there is little or nothing to be seen upon the skin, yet the mother will state that the "rash comes out thick at night." Many of these cases are simply instances of a fleeting erythema associated with errors in feeding, and are of comparatively little consequence. The vaso-motor system, as, indeed, the organs of circulation, should always be examined in cases of this sort, especially in older children, for a persistent erythema of the hands or feet may be due to some cardiac weakness which has remained, perhaps, quite unsuspected. The presence of dermatographia or factitious urticaria is a valuable sign of vaso-motor instability. Finally, the quality and texture of the hair should be noted, for tuberculous affections of the skin are not unfrequently seen in children with fine, silky hair, and long, delicate eyelashes.

